# Hydrostatic pressure-enabled transformation in *Natronomonas pharaonis*: breaking barriers in haloalkaliphilic Archaea genetics

**DOI:** 10.3389/fmicb.2026.1774663

**Published:** 2026-02-20

**Authors:** Emma Bonnaud, Philippe M. Oger, Mathieu Orzalesi, Yoann Louis

**Affiliations:** 1 SEGULA Technologies, Villeurbanne, France; 2 INSA de Lyon, UMR5240 CNRS, Université Claude Bernard Lyon 1, Villeurbanne, France

**Keywords:** genetic, haloalkaliphilic Archaea, hydrostatic pressure, *Natronomonas pharaonis*, transformation

## Abstract

Haloalkaliphilic Archaea that thrive in hypersaline and hyperalkaline environments represent valuable models for fundamental research as well as promising resources for biotechnological applications. However, fully exploiting their potential is hindered by the difficulty of their genetic manipulation, due to the limited availability of genetic tools and the use of a transformation protocol that remain slow and weakly efficient. This limits both industrial exploitation and the study of these organisms. In this study, we describe the development of a new, faster, and more efficient transformation method. It relies on the application of hydrostatic pressure, followed by a rapid return to atmospheric pressure, which generates transient pores in the cell membrane, facilitating the uptake of exogenous DNA. The results demonstrate that applying a pressure of 35 MPa in the presence of 1 M NaCl allows the rapid and efficient generation of transformants in *Natronomonas pharaonis*. This protocol enhances transformation efficiency 6.5-fold while cutting the time required to obtain transformants by 17 days, in contrast to Polyethylene Glycol (PEG)-mediated spheroplast transformation. This new transformation method greatly facilitates the genetic manipulation of *Nmn. pharaonis* (and surely other haloalkaliphilic Archaea that are difficult to transform), thereby opening up new industrial and research applications.

## 1 Introduction

Among Archaea, haloalkaliphilic stand out due to their ability to thrive in hypersaline (10%−35% NaCl) (DasSarma and DasSarma, 2015; Moopantakath et al., 2023) and hyperalkaline environments (pH > 9) (Horikoshi, 1999). This resistance has triggered interest in both fundamental research, as models of adaptation to extreme environments, and applied research for their use as cellular chassis and the stability of their enzymes at high salinity and across a broader pH range (Litchfield, 2011; Munawar and Engel, 2013; Zhang et al., 2018; Moopantakath et al., 2023; Bonnaud et al., 2024).

Genetic tools are key to answering a wide range of biological questions and industrial problematics. Access to robust and versatile genetic systems is therefore critical. While such tools are well-established for halophiles, they remain limited for haloalkaliphilic Archaea. To date, only a single transformation protocol and a few vectors have been reported (Atomi et al., 2012; Mayrhofer-Iro et al., 2013; Orsini et al., 2020; Bonnaud et al., 2024).

The current transformation method relies on Polyethylene Glycol (PEG)-mediated spheroplast transformation. Although this approach is simple, reproducible, and robust in halophilic Archaea (Cline et al., 1989a,b; Cline and Doolittle, 1992), it is still very time-consuming and poorly efficient with some haloalkaliphilic species, particularly *Natronomonas pharaonis*. In our laboratory, its transformation using this protocol results in a transformation efficiency of 4.1 CFU/μg DNA. There is therefore a need for a faster and more effective alternative. Because high salt concentrations are essential to maintain cell integrity, electroporation is not a viable option. Nevertheless, the use of hydrostatic pressure represents a promising alternative, as recently demonstrated by the transfection of difficult-to-modify primary cells (such as embryonic stem cells) (Huang et al., 2024).

This method relies on the temporary application of high hydrostatic pressure, followed by a rapid return to atmospheric pressure. Pressures in the range of 0.1–100 MPa have been shown to transiently alter eukaryotic cell membranes by increasing lipid chain ordering, which reducing membrane fluidity, and altering permeability. Nevertheless, these effects are reversible once atmospheric pressure is restored, allowing membrane permeabilization without causing massive cell death. For instance, exposure to pressures ≤ 100 MPa for 10 min or less did not significantly impact eukaryotic cell viability (Huang et al., 2024).

Here, we present a novel method for the transformation of *Nmn. pharaonis*. This aerobic haloalkaliphilic archaeon grows optimally in 3.5 M NaCl and at pH 8.5 (Falb et al., 2005; Oren, 2006; Gonzalez et al., 2010). Although transformation is possible, it is based on a lengthy and inefficient protocol (Orsini et al., 2020). We therefore have developed a transformation protocol inspired from the hydrostatic-pressure method employed in eukaryotes. Our method uses the coordinated action of hydrostatic-pressure, EDTA, reduced salinity and PEG_600_ to affect the S-layer and permeabilize the plasma membrane. The functionality of this method was validated using the pRo-5 shuttle vector. Compared to the conventional method, this protocol is not only more efficient but also faster, providing a robust platform for genetic manipulation and paving the way for future fundamental research and biotechnological applications.

## 2 Materials and methods

### 2.1 Strains

*Nmn. pharaonis* DSM 2160 was used for all experiments. NVM^+^ rich medium (Witte et al., 1997) and modified DSM 205 medium (casamino acid-based complex medium) (Orsini et al., 2020) were used and were prepared as described in Supplementary material S1.

### 2.2 Preparation of DNA

The pRo-5 shuttle vector was obtained from A. Witte (Department of Microbiology, Immunobiology and Genetics, Max F. Perutz Laboratories, University of Vienna, Vienna, Austria). This vector is 9,030 bp and carries a ColE1 origin of replication and *bla* gene (ampicillin resistance) for replication and selection in *E. coli* and a ΦCH1 replication origin and *gyrB* gene (novobiocin resistance) for replication and selection in halophilic/haloalkaliphilic Archaea). It was isolated from *Escherichia coli* DH5_α_ or GM48 to obtain methylated and unmethylated forms, respectively.

### 2.3 Transformation procedure

#### 2.3.1 Transformation solutions

Buffered solution: 0.5/1/1.5/2 M NaCl; 27 mM KCl; 50 mM Tris-HCl pH8. After autoclaving, it was topped up with 15% sucrose (filter-sterilized) and stored at room temperature (RT).

Unbuffered solution: 0.5/1/1.5/2 M NaCl; 27 mM KCl; Adjust to pH~7. After autoclaving, it was topped up with 15% sucrose (filter-sterilized) and stored at RT.

60% PEG_600_: 60% (v/v) PEG_600_ and 40% (v/v) unbuffered solution. PEG_600_ was stored at −80 °C for up to 6 months and then thawed at 65 °C.

#### 2.3.2 PEG-mediated spheroplast transformation

This protocol was adapted from Orsini et al. (2020). *Nmn. pharaonis* was cultured in modified DSM 205 medium with 70 μg/ml of bacitracin at 37 °C and 110 rpm. After 72 h (OD_600_ 0.2–0.3, ~1–1.5 × 108 cells/ml), the culture was centrifuged for 15 min at 3,900 × g. The pellet was resuspended in buffered solution (2 M) at half the culture volume, containing 0.3 mg/ml pronase E. The cells were then incubated for 48 h at 42 °C. The combination of bacitracin and pronase E enables the removal of the S-layer (a pseudo-crystalline proteinaceous layer that constitutes the archaeal cell wall) and the formation of spheroplasts. After verifying the spheroplastic appearance of the cells by microscopy, 3 ml of spheroplasts were collected by centrifugation (3 min at 10,000 × g) and resuspended in 150 μl of buffered solution (2 M). After adding 50 mM EDTA pH 8 and incubating for 10 min at RT, 5 μg of DNA was added (as used in Derntl et al., 2015) and incubated for 5 min at RT. The cells were then mixed with 150 μl of 60% PEG_600_ [in unbuffered solution (2 M)] and incubated for 30 min at RT. The cells were washed twice with 1 ml of NVM^+^ medium, then centrifuged for 3 min at 10,000 × g. After resuspension of the pellet in 1 ml of NVM^+^ medium, the cells were regenerated at 37 °C and 110 rpm for 48 h. After verification of cell regeneration by microscopy, 100 μl of cells were plated onto NVM^+^ agar plates containing 10 μg/ml of novobiocin. The plates were incubated at 42 °C until colonies appeared. Transformation efficiency was calculated by counting all colonies appearing from the first detectable transformants (about 3 weeks) up to the last colonies observed after 5 weeks of incubation.

#### 2.3.3 Hydrostatic pressure-mediated transformation with PEG_600_ (hydrostatic pressure-based protocol)

**Figure 1:**
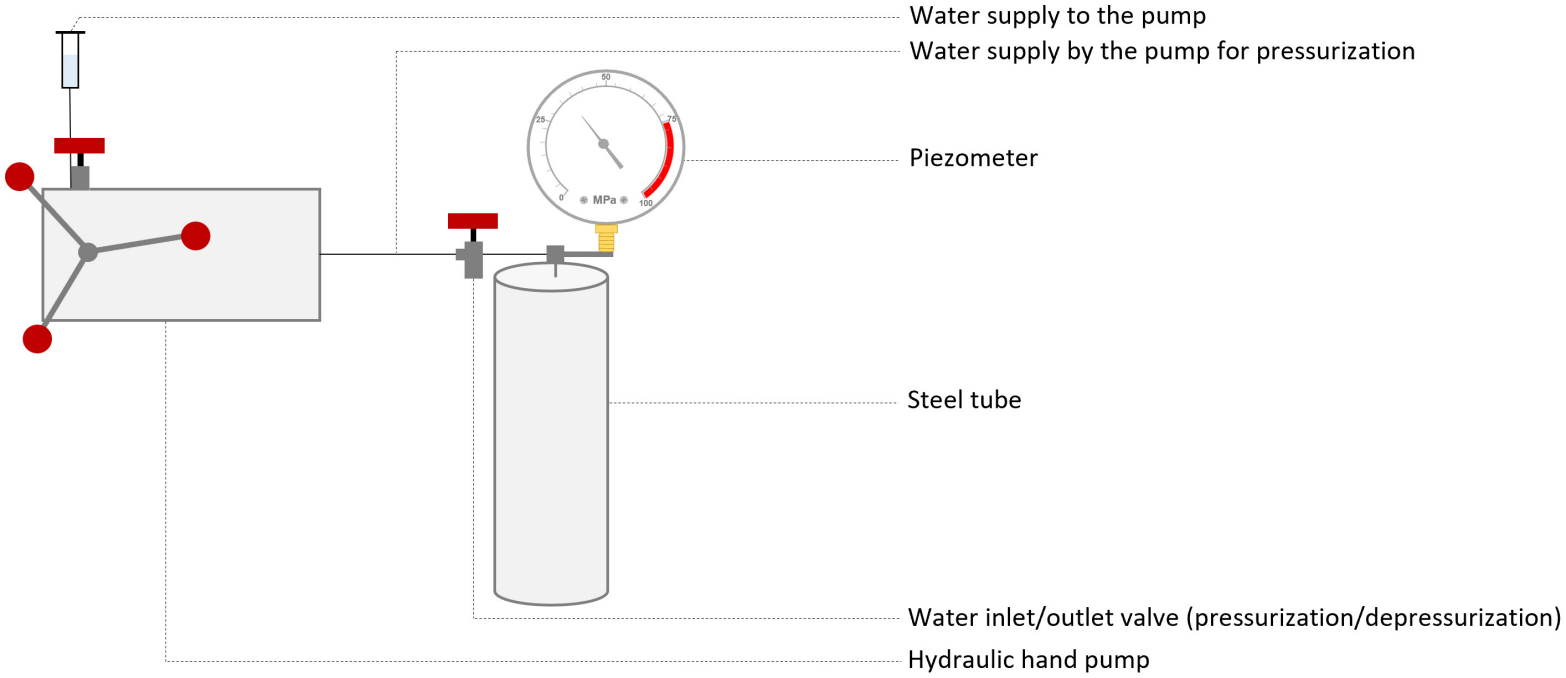
Schematic representation of the hydrostatic pressure system. The samples are placed in syringes, which are then inserted into a steel pipe previously filled with water. Once the water supply to the pump is connected to the hydraulic hand pump, the pressure is increased using the manual pump. The various valves allow access to the water to be opened or closed. To return the pressure to atmospheric level, simply disconnect the water supply to the pump and open the water inlet/outlet valve.

The transformations were performed using cultures of *Nmn. pharaonis* in modified DSM 205 medium. To this end, 3 ml of a culture at the end of its exponential phase (OD_600_ 0.3–0.4, ~1.5–2 × 108 cells/ml) was centrifuged for 3 min at 10,000 x g and the pellet was resuspended in 150 μl of buffered solution (0.5–2 M). After adding 50 mM EDTA pH 8 and incubating for 10 min at RT, 5 μg of DNA was added and incubated for 5 min at RT. The cells were then mixed with 150 μl of 60% PEG_600_ in unbuffered solution (0.5–2 M) and incubated for 30 min at RT. The mixture was then introduced into a 1 ml syringe sealed with a hermetic rubber stopper and placed inside a steel tube. Pressure was applied using a manual hydraulic pump to 10–60 MPa (Figure 1). For the 0 MPa condition, samples were not placed into a syringe or steel tube. The steel tubes had an internal diameter 38 mm, a maximum operating temperature of 250 °C and a maximum pressure of 90 MPa. The target pressure was reached at a rate of 60 MPa per minute. Pressure was released instantly (a few milliseconds), by opening the high pressure valve. Following treatment, cells were washed twice with 1 ml of NVM^+^ medium and centrifuged for 3 min at 10,000 × g. After resuspension of the pellet in 1 ml of NVM^+^ medium, the cells were regenerated at 37 °C and 110 rpm for 48 h. Subsequently, 100 μl of cells were plated onto NVM^+^ agar plates containing 5 or 10 μg/ml of novobiocin. The plates were incubated at 42 °C until colonies appeared. Transformation efficiency was calculated by counting all colonies appearing from the first detectable transformants (which could appear as early as 1 week, depending on the condition) up to the last colonies observed after 5 weeks of incubation.

#### 2.3.4 Hydrostatic pressure-mediated transformation with betaine

The transformations were carried out using cultures of *Nmn. pharaonis* in modified DSM 205 medium. To this end, 3 ml of a culture at the end of its exponential phase (OD_600_ 0.3–0.4, ~1.5–2 × 10^8^ cells/ml) was centrifuged for 3 min at 10,000 × g and the pellet was resuspended in 150 μl of betaine solution (4.3 M betaine in 50 mM Tris-HCl pH 8.7). Next, 5 μg of DNA was added and incubated for 10 min at RT. A pressure of 35 MPa was then applied as described previously (Figure 1). The cells were then washed twice with 1 ml of NVM^+^ medium and centrifuged for 3 min at 10,000 × g. After resuspension of the pellet in 1 ml of NVM^+^ medium, the cells were regenerated at 37 °C and 110 rpm for 48 h. Subsequently, 100 μl of cells were plated onto NVM^+^ agar plates with 10 μg/ml of novobiocin. The plates were incubated at 42 °C until the appearance of transformants. Transformation efficiency was calculated by counting all colonies appearing from the first detectable transformants (about 3 weeks) up to the last colonies observed after 5 weeks of incubation.

### 2.4 Cell viability and regrowth assay

Cell survival and regrowth were assessed using the colony-forming unit (CFU) method described in Oger et al. (2008). WT cells (prior to transformation) and transformants, both before and after regeneration, were serial diluted from 10 to 10^−6^ in NVM^+^ medium without organic compounds. Droplets of 10 μl from each dilution were spotted in quadruplicate onto NVM^+^ agar plates and dried for 30 min. After incubation for 7 days at 42 °C, colonies were counted, and the survival of transformants was compared to that of WT cells.

### 2.5 Screening for positives clones

To identify positive clones, isolated colonies were screened by colony PCR. Each colony was resuspended in 25 μl of sterile dH_2_O and incubated at 95 °C for 10 min. Five microliters of the resulting lysate were used as template for PCR amplification in a 25 μl reaction using GoTaq^®^ Green Master Mix and pEB primers. PCR reactions were performed under the following conditions: initial denaturation at 95 °C for 5 min, followed by 30 cycles of 95 °C for 1 min, 55 °C (annealing temperature) for 1 min and 72 °C for 1 min, with a final extension at 72 °C for 5 min. Primers sequences are as follow, and the expected amplification product was 221 bp.

pEB_Fw: AAACACGCACACCGAAAACG

pEB_Rev: GGAAGAGCCAGGAAACAGCTA

### 2.6 Statistical analysis

All quantitative data were analyzed for statistical significance using R studio (version 4.3.2). A one-way ANOVA was conducted, followed by *post-hoc* comparisons among all group combinations using Tukey's Honestly Significant Difference (HSD) test, with significance defined at α = 0.05. Groups that were not significantly different were assigned the same letter, while groups with statistically significant differences were assigned different letters. All values are presented as mean ± standard deviation.

## 3 Results

### 3.1 Improving PEG-mediated spheroplast transformation by a hydrostatic pressure step

In haloarchaea, the MRR restriction endonuclease targets foreign DNA methylated at GA^m6^TC and CC^m6^GG sites, which are introduced by the Dam and Dcm methylases, respectively (Holmes et al., 1991). As these methylases are present in *E. coli*, plasmid DNA extracted from this bacterium carries these modifications, leading to a strong reduction in transformation efficiency in some species, notably *Haloferax volcanii* (up to a 2,000-fold decrease) (Holmes et al., 1991; Haque et al., 2020). In contrast, as previously observed in the haloalkaliphilic archaeon *Natrialba magadii*, DNA methylation at these sites have no detectable effect on transformation efficiency (Mayrhofer-Iro et al., 2013). In *Nmn. pharaonis*, transformation with methylated and unmethylated plasmids [isolated from *E. coli* GM48 (*dam*^−^/*dcm*^−^)] yielded comparable transformation efficiencies (0.39 and 0.43 log_10_ CFU/μg plasmid DNA for the methylated and unmethylated forms, respectively). This further supports the hypothesis that haloalkaliphilic and halophilic Archaea possess distinct MRR restriction endonucleases and that DNA methylation at these sites does not affect transformation.

In the absence of a methylation effect, an alternative strategy was considered. To improve membrane permeabilization and thus DNA uptake, we used the standard PEG-mediated spheroplast protocol and tested whether a pressurization/depressurization step (P_MPa_ = 35, initial pressure tested) after DNA addition would increase transformation efficiency. Since spheroplasts are fragile due to the loss of their S-layer, we also tested this protocol on intact cells (i.e., using the same protocol but without bacitracin and pronase E for spheroplast formation). Compared with intact cells, which show a 100 % growth recovery, spheroplasts show a markedly reduced ability to recover under these new conditions. Indeed, their growth recovery rate after regeneration decreases significantly to 2.5% (±3.2). One of the four replicates showed 100% survival and was therefore considered an outlier and excluded from the analysis. This unusually high survival likely resulted from incomplete spheroplast formation (observed via microscopy), leaving a larger proportion of intact cells than in the other replicates, which remained potentially transformable under high-pressure treatment.

Regarding transformation efficiency, intact cells yielded 2.2 log_10_ CFU/μg DNA, whereas spheroplasts produced only a single transformant across the four replicates.

Thus, adding a pressurization/depressurization step (P_MPa_= 35) increased the transformation efficiency only with intact cells, from 0.4 (PEG-mediated spheroplast protocol) to 2.2 log_10_ CFU/μg DNA (hydrostatic pressure-based protocol). The formation of spheroplasts is not compatible with the use of hydrostatic pressure for DNA transformation. Indeed, having lost their S-layer, spheroplasts are more fragile, making them more sensitive to the stresses induced by high-pressure treatment. The hydrostatic pressure-based protocol will therefore be carried out using intact cells, which will save 3 days over the total duration of the protocol (spheroplasts formation time minus the culture time required to initiate hydrostatic pressure-based protocol).

### 3.2 Optimization of the Hydrostatic pressure mediated transformation with PEG_600_

After demonstrating that hydrostatic pressure can be used to transform *Nmn. pharaonis*, other parameters were investigated to assess their impact on transformation efficiency and to try to optimize the protocol.

#### 3.2.1 No impact of hydrostatic pressure level on transformation efficiency

Biophysical methods for cellular transformation rely on a delicate balance between inducing sufficient membrane disruption to allow the entry of exogenous DNA and preserving cell viability (Huang et al., 2024). To identify the optimal pressure required to achieve this balance, we tested a range of hydrostatic pressures from 0 to 60 MPa. For each condition, we evaluated both cell survival/regrowth and transformation efficiency (calculated based on all colonies appearing up to 5 weeks of incubation).

As shown in Table 1, applying pressure ranging from 0 to 60 MPa did not significantly affect cell survival prior to regeneration. For most pressures conditions, survival rates remained between 64.4 and 100%, except at 20 MPa, where survival dropped significantly to 25.1% (Table 1). Considerable variability between replicates was also observed for some pressure (Table 1). After regeneration, cell recovery reached 100% across all pressure conditions (Table 1).

**Table 1:** Effect of pressure level on *Nmn. pharaonis* transformation.

**Pressure (MPa)**	**Transformation efficiency (log**_**10**_**(CFU/**μ**g DNA) (**±**SD))**		**Cell survival (%)**	**Post-transformation growth recovery (%)**
	**5** μ**g/ml novobiocin**	**10** μ**g/ml novobiocin**		
PEG-mediated spheroplast protocol	B.D.L	0.4 (± 0.6)^a^	ND	100 (± 0)^a^
0	0.1 (± 0.3)^b^	0.7 (± 1.2)^a^	100 (± 0)^a^	100 (± 0)^a^
10	2.3 (± 0.5)^a^	1.3 (± 1.1)^a^	84.4 (± 0.2)^a^	100 (± 0)^a^
20	2.2 (± 0.1)^a^	2.1 (± 0.3)^a^	25.1 (± 14.3)^b^	100 (± 0)^a^
30	2.2 (± 0.3)^a^	1.4 (± 1.3)^a^	100 (± 0)^a^	100 (± 0)^a^
35	2.6 (± 0.4)^a^	2.2 (± 0.2)^a^	100 (± 0)^a^	100 (± 0)^a^
40	2.4 (± 0.1)^a^	1.3 (± 1.1)^a^	100 (± 0)^a^	100 (± 0)^a^
50	1.9 (± 0.6)^a^	1.8 (± 1.5)^a^	64.4 (± 30.9)^ab^	100 (± 0)^a^
60	1.9 (± 0.3)^a^	1.0 (± 1.0)^a^	83.8 (± 8.1)^a^	100 (± 0)^a^

(**Right**) Effect of pressure level on cell viability before regeneration and on growth recovery after regeneration, relative to the WT prior to transformation. Serial dilutions were spotted onto NVM^+^ agar plates. (**Left**) Variation of transformation efficiency as a function of pressure level. Transformation assays were performed at pressures ranging from 0 to 60 MPa and a salt condition of 2 M.

B.D.L, below the detection limit; ND, not determined.

All experiments were performed in triplicate and results are shown as mean ± SD. Data were analyzed using a one-way ANOVA, followed by Tukey's HSD test. Same letters indicate no significant differences.

As shown in Table 1, cell transformation induced by hydrostatic pressure in the presence of the pRo-5 vector led to the appearance of novobiocin-resistant colonies across the entire range of pressures tested. For pressures ≥10 MPa, transformation efficiencies ranged from 1 to 2.6 log_10_ CFU/μg DNA, with the highest efficiencies observed at 35 and 40 MPa at 5 μg/ml of novobiocin and 20 and 35 MPa at 10 μg/ml of novobiocin (Table 1).

At 5 μg/ml of novobiocin, application of pressure led to a significant increase in transformation efficiency compared with the non-pressurized condition (Table 1). Nevertheless, in the absence of pressure (0 MPa), transformants were detected, but at much lower frequencies for both novobiocin concentrations (Table 1).

Compared to the standard PEG-mediated spheroplast transformation, the hydrostatic pressure-based protocol increased transformation efficiency by 5.5-fold at 10 μg/ml of novobiocin (2.2 vs. 0.4 log_10_ CFU/μg DNA for the hydrostatic pressure-based protocol at 35 MPa and the standard protocol, respectively) and by 6.5-fold at 5 μg/ml of novobiocin (2.6 vs. 0.4 log_10_ CFU/μg DNA for the hydrostatic pressure-based protocol at 35 MPa and the standard protocol, respectively; Table 1).

These results suggest that applying hydrostatic pressure ≥10 MPa probably induces a transient increase in membrane permeability, thereby facilitating DNA uptake. Since the magnitude of the applied pressure applied did not significantly affect transformation efficiency, a pressure of 35 MPa was selected for subsequent experiments, as it provided the best combination of cell survival/regrowth and transformation efficiency.

In the PEG-mediated spheroplast transformation protocol, false positives (typically 1–2 true transformants out of ≤ 10 colonies) were observed at 5 μg/ml novobiocin, whereas none were detected at 10 μg/ml. Therefore, the hydrostatic pressure-based protocol was tested at both concentrations to determine the most suitable condition. Only a few false positives ( ≤ 1% of the clones obtained) were detected for both novobiocin concentrations, with similar frequencies. Due to the higher number of transformants at 5 μg/ml of novobiocin (2.6 log_10_ CFU/μg DNA, which is 6.5-fold higher than the PEG-mediated spheroplast transformation protocol and 1.2-fold higher than the hydrostatic pressure-based protocol at 10 μg/ml of novobiocin; Table 1), this concentration was selected for the final protocol.

In conclusion, the hydrostatic pressure-based protocol (35 MPa with 5 μg/ml novobiocin) enabled successful transformation and increased efficiency up to 6.5-fold compared to the PEG-mediated spheroplast transformation protocol. A pressure of 35 MPa was therefore used for all of the following results.

#### 3.2.2 NaCl concentration modulates the timing of transformants emergence

*Nmn. pharaonis* requires at least 2 M NaCl to maintain cellular integrity (Bonnaud et al., 2024) and this concentration is therefore used as the standard concentration in transformation solutions (Orsini et al., 2020). Here, we investigated whether a further reduction in NaCl concentration (in both buffered and unbuffered solution used for the DNA introduction step) could improve transformation efficiency under the hydrostatic pressure-based protocol by weakening the cell membrane without compromising viability. Four different NaCl concentrations (0.5/1/1.5/2) were tested, and for each condition, both cell survival/regrowth and transformation efficiency (calculated based on all colonies appearing up to 5 weeks of incubation) were assessed.

**Figure 2:**
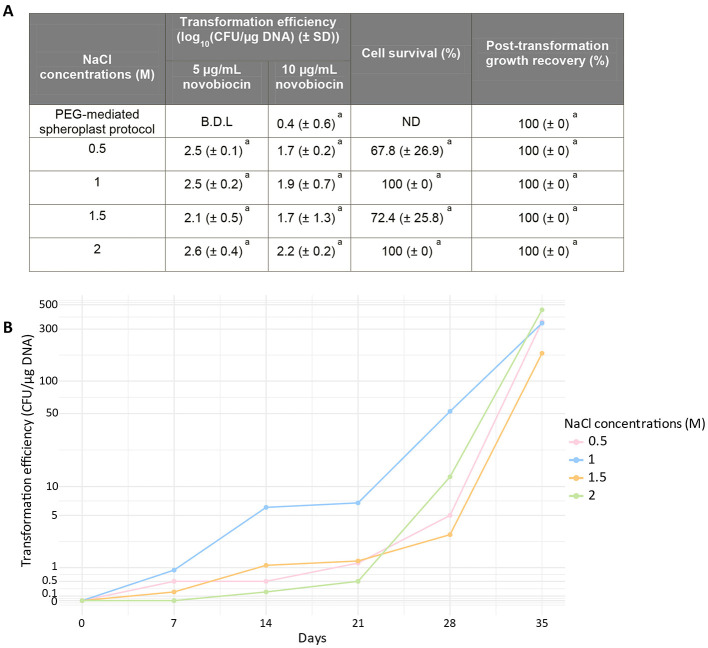
Effect of salt concentration on the transformation of *Nmn. pharaonic*. **(A)** (**Right**) Effect of salt concentration on cell viability before regeneration and on growth recovery after regeneration, relative to the WT prior to transformation. Serial dilutions were spotted onto NVM^+^ agar plates. (**Left**) Variation of transformation efficiency as function of salt concentration. *Condition excluded due to a high number of false positives (typically 1–2 true transformants out of ≤10 colonies). ND, not determined. **(B)** Effect of NaCl concentration on transformation efficiency over time. The mean transformation efficiency is shown at each time point. Transformation assays were performed at NaCl concentrations ranging from 0.5 to 2 M and at a pressure of 35 MPa. All experiments were performed in triplicate. For **(A)**, data are presented as mean ± SD. Statistical analysis was performed using one-way ANOVA followed by Tukey's HSD test. Same letters indicate no significant differences.

Cell survival and regrowth remained high under most conditions (Figure 2A). At the lowest concentration (0.5 M), a non-significant decrease in survival was observed prior to regeneration, with an average survival rate of 67.8% (± 26.9%; Figure 2A). However, following regeneration, 100% of regrowth was observed (Figure 2A). This suggests that although the osmotic downshift to 0.5 M causes substantial initial cell death, the regeneration time allows the surviving population to re-establish a normal phenotype. At 1.5 M, a non-significant decrease is also observed before regeneration, with an average survival rate of 72.4% (±25.8%).

Even with a decrease in survival under some conditions, transformants were obtained at all tested concentrations (Figure 2A). Overall transformation efficiency across all NaCl concentration was around 2.4 log_10_ CFU/μg DNA at 5 μg/ml of novobiocin and 1.9 log_10_ CFU/μg DNA at 10 μg/ml of novobiocin, with no significant differences between concentrations of salt (Figure 2A). The highest transformation measured values were at 2 M NaCl (2.6 log_10_ CFU/μg DNA at 5 μg/ml of novobiocin and 2.2 log_10_ CFU/μg DNA at 10 μg/ml of novobiocin; Figure 2A). Looking at the standard deviations shows that the intervals overlap, indicating that salt concentration has no significant impact on the transformation efficiency (Figure 2A).

The timing of colony appearance varied with NaCl concentration (Figure 2B). Over the first 3 weeks, transformation efficiency was higher at 1 M, with colonies appearing as early as 1 week and reaching an average of 6.8 CFU/μg DNA. In contrast, at 2 M, the first transformants were observed only after 4 weeks (>1 clone), with an average efficiency of 0.5 CFU/μg DNA (Figure 2B). These results indicate that, although overall transformation efficiency over 5 weeks is similar across NaCl concentrations, a transient reduction to 1 M NaCl facilitates earlier clone emergence and higher efficiency during the initial weeks of incubation.

In conclusion, the temporary reduction of the NaCl concentration to 1 M allows the overall efficiency of the transformation to be maintained while promoting faster emergence of transformants and higher efficiency during the first few weeks. This adjustment saves up to 21 days on the total duration of the protocol (first transformants at 7 days at 1 M vs. 28 days at 2 M).

#### 3.2.3 The need of PEG_600_

The PEG-mediated spheroplast transformation method (Orsini et al., 2020) relies partly on PEG_600_, which allows cells to become permeable and facilitates the entry of DNA. To assess whether this compound is essential for the successful generation of transformants under hydrostatic pressure-based protocol conditions, we evaluated the efficiency of the protocol with and without PEG_600_. All transformations were performed under “high-salt” condition (2 M NaCl), i.e., the standard condition. Cells had greater difficulty recovering without PEG_600_. Omission of PEG_600_ reduced post-regeneration growth from 100% (with PEG_600_) to 66.1% (±37.2) but this difference was not statistically significant due to the high variability among replicates.

Regarding transformation efficiency, only one of the four replicates yielded transformants of the same order of magnitude as those obtained with PEG_600_ (2.5 log_10_ CFU/μg DNA without PEG_600_ vs. 2.6 log_10_ CFU/μg DNA with PEG_600_). Since this step is very simple and transformation efficiency shows considerable variability between replicates in absence of PEG_600_, we therefore chose to keep proceeding using PEG_600_ in the final protocol.

#### 3.2.4 Impact of repeated pressure cycles on transformation efficiency

The study by Huang et al. *(*2024) indicated that the duration of the applied pressure does not appear to influence transformation efficiency. We therefore investigated the effect of multiple pressurization/depressurization cycles. Since treatment at 35 MPa did not affect cell survival (Table 1), we aimed to determine whether applying several cycles could enhance transformation efficiency without substantially compromising cell viability.

Before regeneration, cell survival is maintained at 100% for up to two pressurization/depressurization cycles (Table 2). In the third cycle, a marked drop in cell viability is observed (46.7%). However, after four cycles, survival returns to 100% (Table 2). After regeneration, no difference is observed between the different pressure cycles (Table 2). Thus, *Nmn. pharaonis* shows good resistance to repeated cycles of successive pressurization/depressurization, with no significant alteration in survival. Regarding transformation efficiency, no difference was observed between one and multiple cycles (Table 2).

**Table 2:** Effect of pressure cycles on the transformation of *Nmn. pharaonis*.

**Number of cycles**	**Transformation efficiency (log_10_(CFU/μg DNA))** **5 μg/ml novobiocin**	**Cell survival (%)**	**Post-transformation growth recovery (%)**
PEG-mediated spheroplast protocol	B.D.L	ND	100
1	2.6 (±0.4)	100	100
2	1.3	100	100
3	2.1	46.7	100
4	2.6	100	100

(Right) Effect of pressure cycles on cell viability before regeneration and on growth recovery after regeneration, relative to the WT prior to transformation. Serial dilutions were spotted onto NVM^+^ agar plates. (Left) Variation of transformation efficiency with pressure cycle. Transformation assay was performed at a pressure of 35 MPa and at 1 M of NaCl.

B.D.L, below the detection limit; due to a high proportion of false positives (typically 1–2 true transformants out of ≤ 10 colonies). ND, not determined.

#### 3.2.5 The need for cell regeneration

Similar to electroporation, high-pressure-mediated transformation relies theoretically on the transient formation of pores in the cell membrane, followed by their closure and the restoration of membrane integrity (Huang et al., 2024). Although “poration” is transient, we wanted to determine whether a regeneration step was still necessary to obtain transformants.

Regardless of salt concentration, cells show high survival rates of 100% (1 M/2 M at 35 MPa) before and after regeneration (Table 1 and Figure 2). However, no transformant was obtained prior to regeneration. Thus, although “poration” is transient, the recovery step appears to be necessary after high-pressure treatment in order to obtain transformants. This regeneration time is undoubtedly necessary for the cell to restore normal metabolism and express the inserted antibiotic resistance gene.

## 4 Discussion

The transformation of haloalkaliphilic Archaea is currently limited to a protocol based on spheroplast formation and the use of PEG_600_ (Mayrhofer-Iro et al., 2013; Orsini et al., 2020). Although functional, this method remains relatively inefficient (0.4 log_10_ CFU/μg DNA in *Nmn. pharaonis*).

Conventional methods, such as using unmethylated vector, electroporation and lipofection, were tested but proved ineffective (Gulko, 2010). In hard to transform eukaryotic cells, application of a static pressure (60–80 MPa for 30 s), followed by a sudden depressurization, has been reported to enhance transformation efficiency (Huang et al., 2024). Based on this principle, a hydrostatic pressure-based protocol was developed for *Nmn. pharaonis*. In eukaryotes, this step is applied in combination with an electroporation buffer. For haloalkaliphilic and halophilic Archaea, electroporation requires a betaine-based solution to maintain osmolarity while avoiding salt (incompatible with the electric arc). Initial tests combining the betaine buffer with the pressure step yielded 0.7 CFU/μg DNA (Supplementary Table S1). We therefore built upon the PEG-mediated spheroplast transformation protocol, introducing a second level of membrane permeabilization through pressurization/depressurization to enhance its efficiency.

We demonstrated that applying hydrostatic pressure ≥10 MPa, followed by rapid depressurization, significantly enhances transformation efficiency of *Nmn. pharaonis* (0.1 log_10_ CFU/μg DNA at 0 MPa vs. 2.6 log_10_ CFU/μg DNA at 35 MPa, both in the presence of 5 μg/ml of novobiocin; Table 1). This indicates that the high-pressure step facilitates DNA uptake, probably through the transient formation of pores in the membrane. This effect is consistent with the membrane-ordering properties of pressure observed in Archaea and Bacteria (Kish et al., 2012), which can promote transient pore formation. Furthermore, the brutal release of pressure has been previously shown to disrupt membranes in bacteria such as *E. coli* or Eukaryota such as the fission yeast, further increasing the formation of pore in the membrane. The work of Huang et al. (2024) indicates that only the instant release of pressure can increase transformation efficiency, demonstrating that this quick pressure-release driven pore formation is most likely the key parameter in their protocol. The drastic impact of pressure on spheroplasts of *Nmn. pharaonis* in this study also show that the quick-pressure release affects the membrane. It is thus reasonable to assume that in intact cells, the high-pressure release may damage the S-layer and membrane sufficiently to allow pore formation in the membrane. Furthermore, the number of transformants obtained under hydrostatic pressure-based protocol (P_MPa_ = 35 and 5 μg/ml novobiocin) is higher than that observed with the PEG-mediated spheroplast transformation protocol, resulting in a 6.5-fold increase in transformation efficiency (Table 1). Similar to conventional biophysical methods such as electroporation, this method does not appear to induce irreversible cellular damage, as demonstrated by cell survival before and after regeneration (Table 1). This resistance to high-pressure can be primarily attributed to the structure of the cell envelope in haloalkaliphiles and halophiles (Kish et al., 2012). In particular, the presence of an S-layer has been shown to contribute to the resistance of the cell membrane under pressure, reinforcing membrane integrity in such conditions (Schuster and Sleytr, 2002). Additionally, the presence of intracellular salts may function similarly to piezolytes, enhancing protein stability under high-pressure (Kish et al., 2012). High-salt concentrations also reduce water activity, a mechanism that has been demonstrated to stabilize proteins in high-pressure environments (Oliveira et al., 1994; Smolin and Winter, 2008; Hayman et al., 2008).

Unexpectedly, transformants were obtained without spheroplasts formation and pressure step, although the transformation efficiency was very low (0 MPa, Table 1). This suggests that while pronase E and bacitracin are usually required to remove S-layer and efficiently generate spheroplasts (Gulko, 2010; Mayrhofer-Iro et al., 2013; Orsini et al., 2020), treatment with EDTA alone is probably sufficient to affect S-layer and enable DNA uptake. EDTA alone does not induce the formation of spheroplasts in haloalkaliphilic Archaea (Mayrhofer-Iro et al., 2013) but rather causes the formation of areas of fragility in the S-layer. Microscopic observations following a hydrostatic-pressure step at 35 MPa revealed that the cells retained their normal rod-shape, with no spheroplasts detected (data not shown).

Regarding spheroplast formation, they are too fragile to withstand the pressurization/depressurization step. Furthermore, our results demonstrate that using intact cells (i.e., using the same protocol but without bacitracin and pronase E for spheroplast formation) can yield transformants. This step requires 5 days to complete, so substituting it with a 2-day culture reduces the protocol by 3 days, a significant advantage given its already lengthy duration.

In terms of NaCl concentration, a transient decrease (during DNA introduction) did not significantly affect cell survival before regeneration, nor growth recovery thereafter (Figure 2A). This is likely due to the temporary reduction and the fact that the NaCl concentration remained high enough to prevent osmotic shock or cell lysis. Similarly, no significant variation in final transformation efficiency was detected (Figure 2A). Interestingly, reducing the NaCl concentration to 1 M maintains transformation efficiency while accelerating the appearance of transformants. At this concentration, the first transformants appeared in just 7 days (vs. 28 days at 2 M; Figure 2B), shortening the overall protocol by up to 21 days and enhancing early-stage transformation efficiency (Figure 2B).

PEG_600_ is commonly used to permeabilize the cell membrane in haloalkaliphilic and halophilic Archaea. In this new protocol, an additional permeabilization step involving pressurization and depressurization was introduced. However, PEG_600_ remains essential for efficient membrane permeabilization. Although transformants were obtained in one replicate without PEG_600_, no clones were detected in the other three replicates. These results suggest that PEG_600_ likely plays a key role in promoting membrane permeabilization.

Since the pressurization/depressurization stage (P_MPa_: 35) had no impact on cell survival (Table 1), we hypothesized that more extreme conditions (longer pressurization duration or an increase in the number of pressurization/depressurization cycles) could improve transformation efficiency. The idea is that these conditions could have a greater impact on the membrane, promoting DNA entry. In eukaryotes, it has been shown that the duration of pressurization does not affect transformation efficiency (Huang et al., 2024). Furthermore, haloarchaea are particularly resistant to the application of high-pressures over long periods, as demonstrated by the resistance of *Halobacterium salinarum* NRC1 to 400 MPa for 1 h (85% survival rate) (Kish et al., 2012). We therefore wanted to explore the effect of several pressurization/depressurization cycles. It has been found that *Nmn. pharaonis* appears to tolerate repeated cycles (up to four) well, with no major alteration in survival before regeneration and growth after regeneration (Table 2). Nevertheless, the drop in survival observed at three cycles could be explained by the membrane reaching a critical threshold, causing it to open and leading to a drop in viability. Nevertheless, after this transition, the membrane adopts a more stable state, allowing it to survive beyond three cycles (Table 2). In line with the almost total maintenance of viability, no increase in transformation efficiency was observed (Table 2).

Since spheroplast formation is not required, we wanted to determine whether the regeneration step is essential in this protocol. No transformants were obtained before this step. Although pore formation is normally transient and does not appear to significantly affect cell survival, regeneration seems necessary for restoring a wild-type phenotype and enabling the expression of novobiocin resistance.

With this new protocol (P_MPa_ = 35; 5 μg/ml of novobiocin), the transformation efficiency results are significantly higher than those obtained with the PEG-mediated spheroplast transformation protocol (2.6 vs. 0.4 log_10_ CFU/μg DNA). However, we observed that, unlike the latter, a few false positives could be obtained ( ≤ 1%). This can be explained by the fact that novobiocin is “bacteriostatic” for *Nmn. pharaonis* (Gulko, 2010 and data not shown here). Residual growth may then appear after a certain period of time or when the cell concentration is high. The absence of spheroplast formation leads to a higher cell density, which increases the likelihood of observing such residual growth.

**Figure 3:**
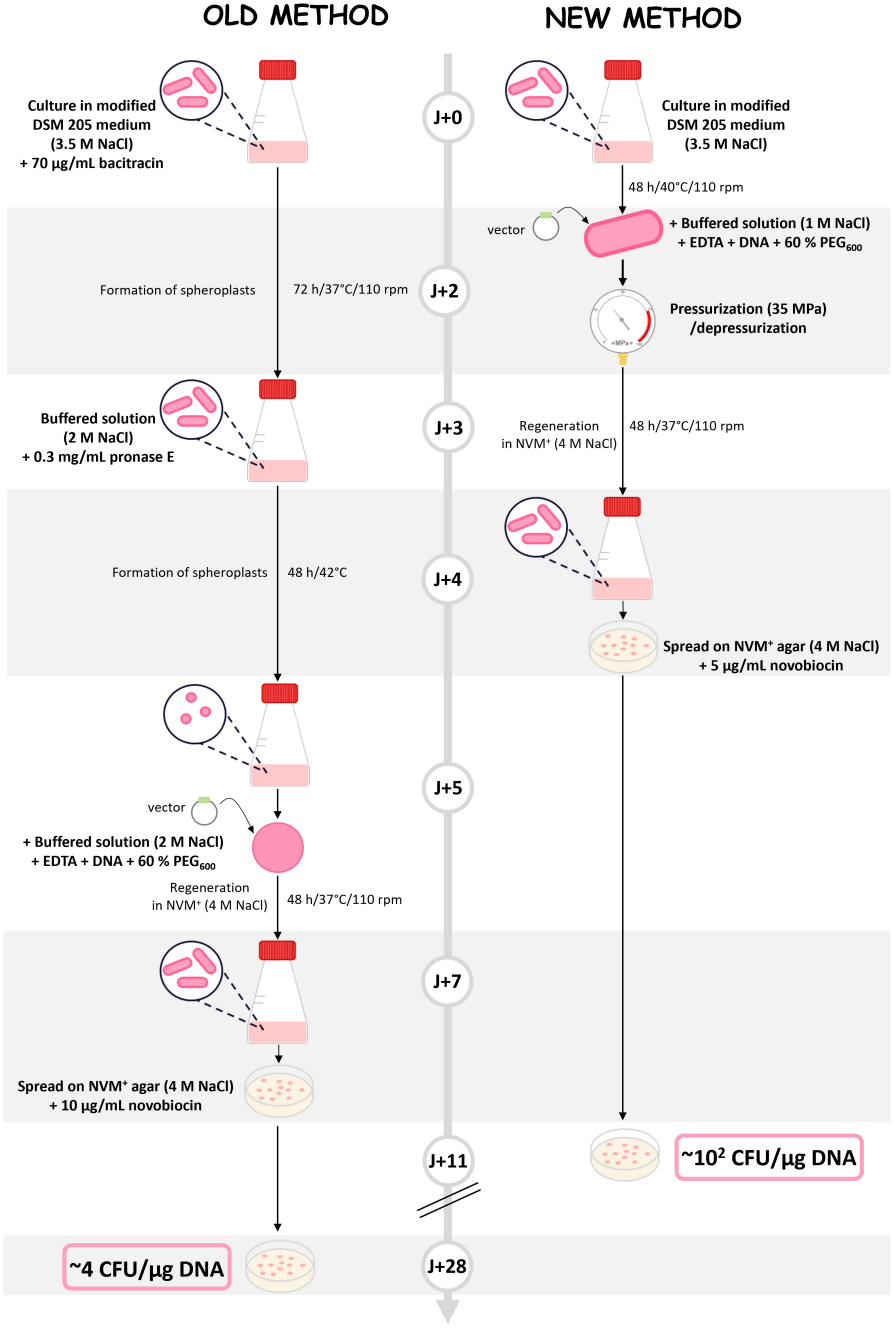
PEG-mediated spheroplast transformation vs. hydrostatic pressure-based protocol. Comparison of the PEG-mediated spheroplast transformation (old method; Orsini et al., 2020) with the hydrostatic pressure-based protocol (new method).

These findings led to the development of the following protocol (Figure 3). Cell transformation is initiated using an intact culture of *Nmn. pharaonis*. The cells are then treated with EDTA and PEG_600_ to affect the S-layer and permeabilize the plasma membrane. After adding the PEG_600_, a second permeabilization step is induced by rapidly pressurizing and depressurizing (P_MPa_ = 35), all of that concomitant with a decrease in salinity to 1 M. The transformed cells are then regenerated for 48 h in a nutrient-rich medium containing 4 M NaCl. The cells are then spread on the same medium with a selection pressure (5 μg/ml novobiocin). Compared to the standard PEG-mediated spheroplast transformation protocol, this approach reduces the total duration by 17 days and increases transformation efficiency by 6.5-fold, greatly facilitating the handling of haloalkaliphilic Archaea.

This new transformation protocol is therefore expected to greatly facilitate the manipulation of *Nmn. pharaonis* and haloalkaliphilic Archaea by providing a quick and efficient method for DNA introduction. The availability of this protocol opens up several key applications: the development of cellular chassis for the production of industrially relevant proteins and functional studies of haloalkaliphilic Archaea through targeted mutagenesis.

## Data Availability

The original contributions presented in the study are included in the article/supplementary material, further inquiries can be directed to the corresponding authors.
