# Supplementary material for: Hydrostatic pressure-enabled transformation in Natronomonas pharaonis: breaking barriers in haloalkaliphilic Archaea genetics
Source: Front Microbiol. 2026 Feb 20;17:1774663. doi: 10.3389/fmicb.2026.1774663 (PMC12963326; doi:10.3389/fmicb.2026.1774663)
Supplement: Supplementary file 1 [file Supplementary_file_1.docx]

Supplementary Material

Supplementary data 1. Media for *Nmn. pharaonis*

- Recipe for NVM^+^ medium:

The composition of 1 L of the medium is as follows:

Casamino acids............................8.8 g

Yeast extract ..............................11.7 g

Tri-Na citrate dihydrate..............3 mM

KCl .......................................31.5 mM

NaCl..............................................4 M

Agar.………………………….…10 g

The pH of the culture medium was adjusted to 9 with 10 N NaOH, then the volume was adjusted to 933 mL with dH2O. After autoclaving for 20 minutes at 121°C, the medium was supplemented with:

Na_2_CO_3_.......................37 mM (dissolved in _d_H_2_O and sterilized by filtration)

MgSO_4_ ....................1 mM (dissolved in _d_H_2_O and sterilized by autoclaving)

FeSO_4_ ......................0.02 µM (dissolved in _d_H_2_O and sterilized by filtration)

- Recipe for modified DSM 205 medium:

The composition of 1 L of the medium is as follows:

Casamino acids..................................15 g

Tri-Na citrate..............................10.2 mM

Glutamic acid…….....................16.9 mM

MgSO_4_ ......................................10.1 mM

KCl.............................................26.8 mM

NaCl................................................3.5 M

The volume of the medium was adjusted to 988 mL with _d_H_2_O and autoclaved for 20 minutes at 121°C. The pH of the medium was adjusted to 8.5 with 12 mL of 20% Na_2_CO_3_.

Supplementary Table 1. Hydrostatic pressure mediated transformation with betaine.

|  | **Transformation efficiency**  (CFU/µg DNA) (± SD)) | **Cell survival (%)** | **Post-transformation growth recovery (%)** |
| --- | --- | --- | --- |
| PEG-mediated spheroplast protocol | 4.1 (±4) ^a^ | ND | 100 (±0) ^a^ |
| Betaine | 0.7 (±0.7) ^a^ | 100 (±0) ^a^ | 100 (±0) ^a^ |
| High-pressure protocol | 342 (±168.1) ^b^ | 93.1 (±7.2) ^a^ | 100 (±0) ^a^ |

(Right) Effect of betaine solution on cell viability before and growth recovery after regeneration. Cell survival and growth recovery assays were performed to evaluate the impact of betaine solution. The number of cells was counted before and after regeneration and was compared to the number of WT cells prior to transformation. Serial dilutions of non-regenerated and regenerated cultures were prepared and spotted onto NVM^+^ agar plates. (Left) Effect of betaine solution on transformation efficiency*.* Each condition was performed in triplicate. Results are shown as mean ± SD and same letters indicate no significant difference using Tukey HSD.
